# Synthesis and decoding of selenocysteine and human health

**DOI:** 10.3325/cmj.2012.53.535

**Published:** 2012-12

**Authors:** Rachel L. Schmidt, Miljan Simonović

**Affiliations:** Department of Biochemistry and Molecular Genetics, University of Illinois at Chicago, Chicago, Ill, USA.

## Abstract

Selenocysteine, the 21st amino acid, has been found in 25 human selenoproteins and selenoenzymes important for fundamental cellular processes ranging from selenium homeostasis maintenance to the regulation of the overall metabolic rate. In all organisms that contain selenocysteine, both the synthesis of selenocysteine and its incorporation into a selenoprotein requires an elaborate synthetic and translational apparatus, which does not resemble the canonical enzymatic system employed for the 20 standard amino acids. In humans, three synthetic enzymes, a specialized elongation factor, an accessory protein factor, two catabolic enzymes, a tRNA, and a stem-loop structure in the selenoprotein mRNA are critical for ensuring that only selenocysteine is attached to selenocysteine tRNA and that only selenocysteine is inserted into the nascent polypeptide in response to a context-dependent UGA codon. The abnormal selenium homeostasis and mutations in selenoprotein genes have been causatively linked to a variety of human diseases, which, in turn, sparked a renewed interest in utilizing selenium as the dietary supplement to either prevent or remedy pathologic conditions. In contrast, the importance of the components of the selenocysteine-synthetic machinery for human health is less clear. Emerging evidence suggests that enzymes responsible for selenocysteine formation and decoding the selenocysteine UGA codon, which by extension are critical for synthesis of the entire selenoproteome, are essential for the development and health of the human organism.

A Swedish chemist, Jöns Jacob Berzelius, isolated selenium from the lead chambers in a sulfuric acid factory in 1817. Because of its similarities to tellurium, which was previously named after the Roman god of earth Tellus (1), Berzelius decided to name the newly discovered element after Selene, the Hellenic moon goddess (2). The unique properties of selenium prompted its wide use in engineering, chemical industry, and glass manufacturing. It is this use that revealed the poisonous properties of selenium, which caused frequent poisoning and selenium-induced deaths in industrial workers (3,4). Reports that selenium is toxic to farm animals (5-7), that it has teratogenic effects in birds (8), non-human primates and man (9-11), and that its high doses might also be carcinogenic (11) further strengthened the notion that selenium is toxic to any living organism. This view held firm until the mid-1950s when it was first reported that trace amounts of selenium (and molybdate) are required for optimal enzymatic properties of intestinal *Escherichia coli* (12) and that selenium is essential for rodent survival (13). This was followed by a series of observations that selenium deficiency is a cause of a variety of livestock diseases such as white muscle disease in cattle and sheep (14,15), exudative diathesis in chicken (16,17), male infertility in mammals (18-20), and mulberry heart disease in pigs (21). In spite of these reports, it was not until the early 1980s that selenium was considered beneficial for humans. The anonymous report that the cardiovascular disease, known as Keshan disease, is caused by selenium deficiency (22) and epidemiological studies on the effect of selenium on cancer and cardiovascular diseases (23), Kashin-Beck disease (24), and myxoedematic cretinism (25) marked the turn of the tide for the field of selenium biology.

Concurrently with the reports on its beneficial role, observations that selenium is a constitutive component of mammalian glutathione peroxidase (GPx) (26-32) and other microbial enzymes such as protein A component of the clostridial glycine reductase (33), formate dehydrogenase (34,35), nicotinic acid hydroxylase, and xanthine dehydrogenase (36) have been published. It took several years of tedious work to show that selenium is present in those and other selenoproteins as the newly identified amino acid – selenocysteine (37-40). Initially, it was thought that selenium is incorporated post-translationally into certain enzymes and proteins, though the mechanism by which this could have taken place was not proposed. Almost a decade later, by a combination of macromolecular x-ray crystallography (41), and protein and gene sequencing (42,43) it was convincingly shown that the selenocysteine residue in the active site of the murine GPx is encoded by a UGA codon (43-45). It is now known that the human selenoproteome comprises 25 selenoproteins that are expressed in various tissues and organs (Table 1) (46). Some selenoproteins are ubiquitously expressed, whereas the others have more restricted tissue and organ distribution. Also, while the biological role of certain selenoproteins is well established, there are still those for which the physiological and cellular role is less clear. With the notable exception of selenoprotein P (SelP), selenoproteins typically contain a single selenocysteine residue that is critical for their structure and function. That selenoproteins are essential for life has been convincingly shown by an embryonically lethal phenotype of the mouse tRNA^Sec^ knock out mutant (47). Furthermore, emerging studies have just began to unravel all the ways selenoproteins affect cell signaling cascades and cell cycle, protein and RNA expression, and disease development in humans. This picture is growing into a complex mosaic, which stands in contrast to an earlier assumption that selenoenzymes and selenoproteins simply protect the cell from oxidative stress and thus play a preventive role against disease development.

**Table 1:** Human selenoproteins, physiological role, and impact on human health

Name	Protein family	Physiological role	Role in human health	Special notes
**GpX1-4, GpX6**	Glutathione peroxidase	Catalyzes the reduction of hydrogen peroxide and/or lipid peroxides. First line of defense against oxidative stress.	Plays a role in defense against cancer, cardiovascular and neurodegenerative disease.	GpX1 – first identified selenoprotein.
**Txnrd1-3**	Thioredoxin reductases	Catalyzes the reduction of oxidized thioredoxin (Trx). Regulate various signaling cascades.	Important for cancer progression and viral suppression.	Txnrd1 and 2 – Housekeeping proteins Txnrd3 – Expressed in testes
**DIO 1-3**	Iodothyronine deiodinases	Membrane-anchored selenoenzymes that activate/inactivate thyroid hormone.	Important for development and regulating overall metabolic rate.	Stable mRNA under conditions of low selenium – suggests a high place in selenoprotein expression hierarchy.
**SelH**	Thioredoxin fold-like protein	Regulates expression of enzymes involved in glutathione synthesis.	Not known	Widely distributed in various tissues.
**SelM, Sep15**	Thioredoxin fold-like protein	Thiol-disulfide oxidoreductases that play a role in protein folding quality control.	Not known	Localized to the endoplasmic reticulum (ER).
**SelT**	Thioredoxin fold-like protein	Not known	Not known	Localized to the ER membrane.
**SelV**	Thioredoxin fold-like protein	Not known	Not known	Expressed in testes.
**SelW**	Thioredoxin fold-like protein	Interacts with glutathione and protein 14-3-3.	Potential antioxidant role.	Expressed in all tissues.
**SelI**	Seven transmembrane domains and a CDP-alcohol phosphatidyltransferase motif	Involved in phospholipid synthesis	Not known	Perhaps localized to the ER.
**SelK**	Integral membrane protein	Not known	Not known	Localized to the ER. High expression in the heart.
**SelS**	Integral membrane protein	Responsible for removal of misfolded proteins, protection from oxidative damage and ER stressed induced apoptosis.	Mutations linked to cancer, cardiovascular disease, preeclampsia and rheumathoid arthritis.	Localized to the ER and plasma membranes.
**SelN (SepN)**	Integral membrane protein	Function in calcium mobilization by direct modulation of the ryanodine receptor.	Mutations linked to multiple muscle system disorders including muscular dystrophy and multiminicore disease.	Localized to the ER. Mutations in the 3′UTR of SelN led to identification of the Sec redefinition element (SRE).
**SelP**		Mainly responsible for selenium transport. Addition functions include glutathione peroxidase activity and heparin and heavy metal binding.	SelP deficiency affects brain and testes, to a lesser extent, heart and kidneys.	Only selenoprotein containing multiple selenocysteine residues. Accounts for 40%-50% of the total selenium in plasma.
**SelR**	Methionine sulfoxide reductase	Reduction of R-form of methionine sulfoxides, oxidized methionines.	Plays a role in protection from neurodegeneration, maintaining lens cell viability, and reducing oxidative damage during aging.	
**SPS2**	Selenophosphate synthetase	Converts selenide into selenophasphate for Sec synthesis.	Not known	Only selenoenzyme involved in selenoprotein synthesis.
**SelO**	Contains Cys-X-X-Sec motif	Not known	Not known	

The observation that selenocysteine is a constitutive component of enzymes immediately raised a series of questions that intrigues the scientific community to the present day: 1) How is selenocysteine synthesized? 2) Does it require a special tRNA to be incorporated into proteins? and 3) Which mechanism distinguishes the selenocysteine UGA codon from the typical translational stop UGA codon? Here, major discoveries in the field of selenocysteine and selenoprotein synthesis, which provided some of the answers to these fundamental questions, will be summarized. Additionally, the most recent clinical observations suggesting that the integrity of the selenocysteine-synthetic and translational machinery is of great importance for human health will be discussed.

## The mechanism of selenocysteine synthesis

**Figure 1:**
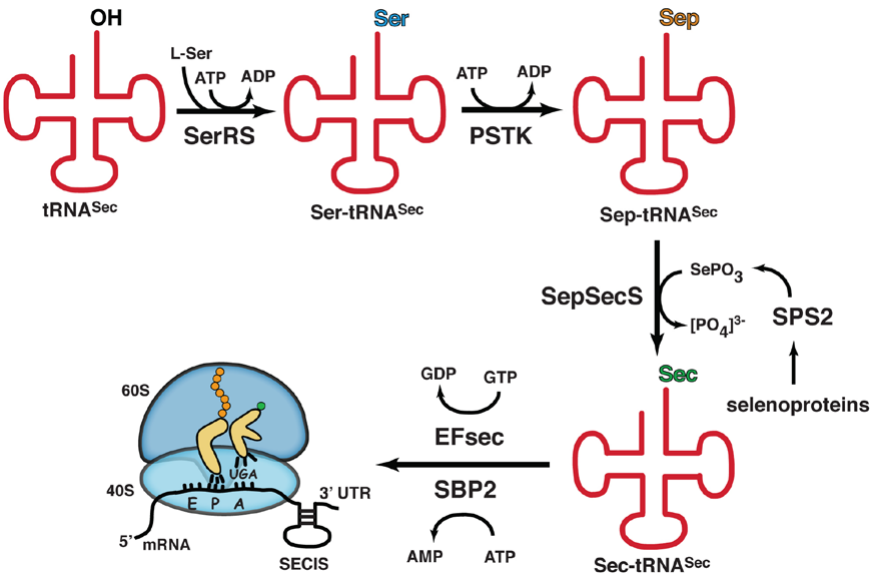
Synthesis and co-translational incorporation of selenocysteine in humans. The cycle, which is conserved in archaea and eukaryotes, begins with a mischarging reaction in which seryl-tRNA synthetase attaches L-serine (L-Ser) to a non-cognate tRNA^Sec^. A specific kinase, O-phosphoseryl-tRNA^Sec^ kinase (PSTK), phosphorylates the seryl group yielding a phosphoseryl (Sep)-tRNA^Sec^ intermediate. In the terminal synthetic reaction, O-phosphoseryl-tRNA^Sec^:selenocysteinyl-tRNA^Sec^ synthase (SepSecS), catalyzes conversion of Sep-tRNA^Sec^ into selenocysteinyl (Sec)-tRNA^Sec^ by a mechanism that requires selenophosphate and a co-factor pyridoxal phosphate (PLP). Selenophosphate, the main selenium donor in man, is a product of the catalytic activity of selenophosphate synthetase (SPS2). Human SPS2 is a selenoenzymes that utilizes as a reaction substrate the final product of selenoprotein/selenocysteine degradation, selenide, and adenosine triphosphate (ATP). Finally, Sec-tRNA^Sec^ is targeted and delivered to the ribosome by a specialized elongation factor – EFsec. An auxiliary protein factor, SECIS-binding protein 2 (SBP2), is required for decoding of the selenocysteine UGA codon in all vertebrates, whereas a shorter ortholog is functional in invertebrates. Selenocysteine (green sphere) is inserted into the nascent protein (orange spheres) in response to a specific UGA codon. SECIS, an *in-cis* element in the selenoprotein mRNA located in the 3′-UTR, forms a stem loop structure and is required for decoding of the selenocysteine UGA codon. In bacteria, a single enzyme, SelA, converts Ser-tRNA^Sec^ to Sec-tRNA^Sec^, elongation factor SelB binds directly to SECIS, which is, in turn, a part of the coding sequence.

Selenocysteine, also known as the 21st amino acid, is unique among the proteinogenic amino acids. It is the only amino acid containing an essential dietary micronutrient (selenium) as a constitutive component, the only amino acid encoded by a UGA codon and the only one synthesized on its tRNA in all domains of life. Moreover, selenocysteine is the only amino acid among over 140 amino acids found in proteins thus far (this count includes 20 standard amino acids, pyrollysine and all post-translationally modified amino acids), which requires a complex tRNA-dependent synthetic machinery for its synthesis, delivery to the ribosome, and insertion into the nascent selenoprotein (Figure 1). Studies on the bacterial selenocysteine pathway in the early 1990s (48) paved the way toward better understanding of the analogous system in archaea and eukaryotes.

In a typical case involving any of the 20 standard amino acids and pyrollysine, an amino acid is formed in the cytosol and then coupled to a cognate tRNA by a specific aminoacyl-tRNA synthetase. Selenocysteine, however, defies the canonical pathway; its synthesis is reminiscent of but more complex than the indirect aminoacylation pathway of asparagine and glutamine found in some archaea. First, a cellular pool of free selenocysteine does not exist, and second, because putative selenocycteinyl-tRNA synthetase never evolved, free selenocysteine, even if it were available, could not be attached to the cognate tRNA. So, how is selenocysteine synthesized and how is it paired with its tRNA? In all selenocysteine-containing organisms, the synthetic cycle of selenocysteine begins with an essential “error:” seryl-tRNA synthetase (SerRS) “erroneously” charges selenocysteine tRNA (tRNA^Sec^) with serine thus yielding seryl-tRNA^Sec^ (Figure 1). The mischarged Ser-tRNA^Sec^ is not edited and is released into solution to serve as an intermediate for the subsequent enzymatic reactions. The ability of SerRS to aminoacylate two tRNAs with completely different anticodon sequences is quite peculiar. It is even more puzzling that SerRS is capable of acting on both tRNA^Ser^ and tRNA^Sec^ with significant efficiency considering that the two tRNAs adopt completely distinct folds. While tRNA^Ser^ is a canonical elongator tRNA that adopts a 7/5 fold (where 7 and 5 indicate a number of base pairs in the acceptor and TΨC arms, respectively), tRNA^Sec^ is an unusual tRNA that adopts either a 9/4 fold in eukaryotes (49,50) or an 8/5 fold in prokaryotes (51). In either case, the acceptor-TΨC helix of tRNA^Sec^ contains 13 base pairs as opposed to a standard length of 12 base pairs observed in all other tRNAs. This difference in the acceptor arm length should impose significant structural and spatial constraints on the interactions between tRNA^Sec^ and selenocysteine-synthetic enzymes. For instance, all tRNA-binding enzymes contain a 5′-phosphate binding groove, among other recognition motifs, which accommodates the 5′-end of the substrate tRNA. Because of the additional base pair in the acceptor arm, the 5′-phosphate in tRNA^Sec^ is translated by ~ 3.4Ĺ and rotated by ~ 33° clockwise around the helix-axis (when viewed down the helix and toward the 3′ and 5′ ends) relative to the corresponding group in canonical tRNAs. However, SerRS is capable of binding tRNAs in which the 5′ phosphate and perhaps other structural elements are positioned quite differently. From the structure of the T. thermophilus SerRS-tRNA^Ser^ complex it is evident that the enzyme binds the elbow of the acceptor-TΨC ‘helix’ of tRNA^Ser^ with its N-terminal domain (52). However, large parts of the acceptor and variable arms were disordered in the crystal and it remains obscure how SerRS interacts with tRNA^Sec^ (52). Further mechanistic and structural studies on the mechanism of substrate promiscuity of SerRS will perhaps explain why this enzyme is capable of “charging” two structurally different tRNAs. Also, it is not clear how frequently human SerRS misacylates tRNA^Sec^ under physiological conditions, whether this process is regulated and how putative regulatory processes might be affecting synthesis of selenocysteine in particular, and selenoproteins in general. Interestingly, the serylation of tRNA^Sec^ is the only reaction in the cycle of selenocysteine that is conserved in all domains of life; beyond this point the bacterial mechanism significantly diverges from the archaeal and eukaryotic processes.

**Figure 2:**
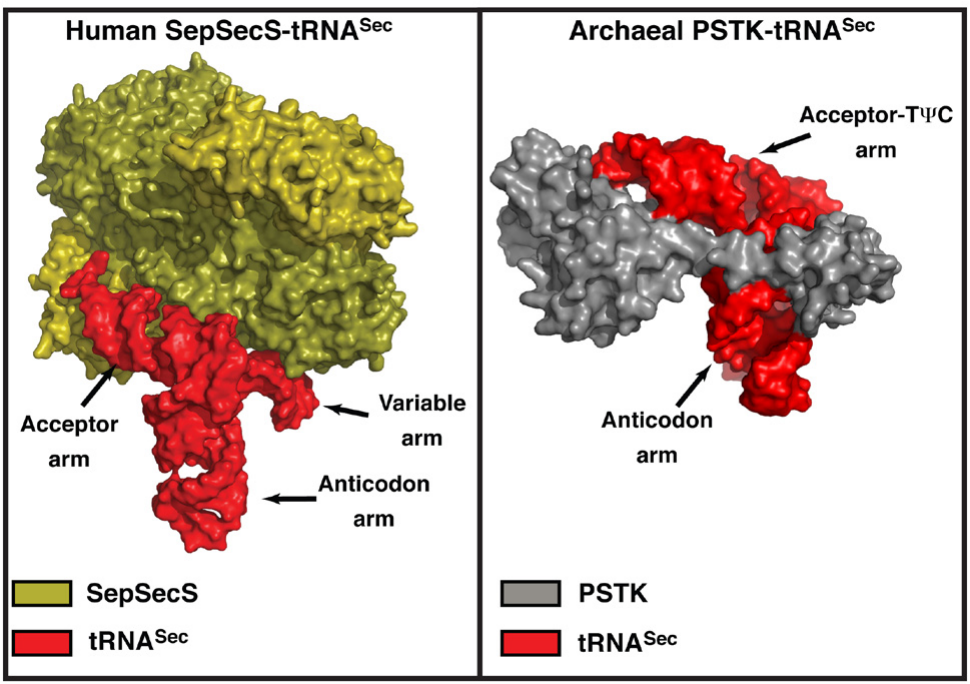
PSTK and SepSecS recognize the distinct fold of tRNA^Sec^ by binding to different structural elements in tRNA^Sec^ (**Left**). Surface diagram of the human SepSecS tetramer (olive) complexed with human tRNA^Sec^ (red) shows that SepSecS binds the major groove of the extended acceptor-TΨC “helix” (based on PDB ID: 3HL2) (49) and it “measures” the distance between the variable arm and the CCA end. (**Right**) Archaeal PSTK (gray) binds the opposite side of tRNA^Sec^ (red) and ‘measures’ the distance between a longer D arm and the CCA end (based on PDB ID: 3ADD) (51). Acronyms explained in Figure 1 legend.

While a single bacterial enzyme, the homodecameric SelA, catalyzes the conversion of Ser-tRNA^Sec^ into Sec-tRNA^Sec^ (53), two enzymatic steps are needed to complete the conversion in archaea and eukaryotes. In the first step, O-phosphoseryl-tRNA^Sec^ kinase (PSTK), phosphorylates Ser-tRNA^Sec^ into phosphoseryl-tRNA^Sec^ (Sep-tRNA^Sec^) (54,55), and in the second, O-phosphoseryl-tRNA^Sec^:selenocysteinyl-tRNA^Sec^ synthase (SepSecS) substitutes phosphate with selenium thus yielding Sec-tRNA^Sec^ (Figure 1) (56-58). In striking contrast to a promiscuous SerRS, PSTK and SepSecS have stringent substrate specificities: PSTK acts on Ser-tRNA^Sec^ and not on Ser-tRNA^Ser^, whereas SepSecS acts only on Sep-tRNA^Sec^ and not on Ser-tRNA^Sec^ or Ser-tRNA^Ser^. Biochemical, biophysical and x-ray crystallographic studies have provided a structural basis for the substrate specificity of these enzymes (Figure 2).

Identification of the bacterial SelA as the enzyme that directly converts serine to selenocysteine in a tRNA-dependent manner stimulated a search for archaeal and eukaryotic homologs. However, all efforts to identify a SelA-like enzyme in these organisms failed. Ultimately, it was studies published in the early 1980s by Hatfield et al that paved the way toward the solution for the conundrum (59,60). The authors have characterized a distinct opal (UGA) suppressor tRNA isolated from bovine liver that could suppress the termination of protein synthesis at the UGA codon, that carried an unusual phosphoseryl group attached to its 3′ end and that it participated in the phosphorylation reaction. Two decades later, a tRNA-dependent kinase responsible for formation of Sep-tRNA^Sec^ was identified as PSTK (54,55). An intriguing question is what would be the purpose of the phosphorylation event. The simplest explanation is that Sep-tRNA^Sec^ intermediate contains phosphate in its aminoacyl moiety, which is a better leaving group than water. In other words, phosphorylation activates the β-OH group of serine for a subsequent substitution with selenium. Also, insertion of the tRNA-dependent kinase adds yet another checkpoint that ensures proper decoding of an in-frame selenocysteine UGA codon. The crystal structure of the archaeal PSTK-tRNA^Sec^ binary complex revealed that the dimeric PSTK recognizes the distinct 8/5 fold of the archaeal tRNA^Sec^ by acting as a molecular ruler that “measures” the distance between the enlarged D arm and the tip of the long acceptor arm of tRNA^Sec^ (Figure 2) (51). The kinase interacts with the upper body of tRNA and it does not interact with the anticodon arm or anticodon sequence, which is a trademark of all enzymes involved in selenocysteine synthesis and insertion (ie, SerRS, SepSecS, and EFsec). Biochemical assays have confirmed the mode of tRNA^Sec^ recognition utilized by the archaeal PSTK (51,61) and by extension it was proposed that the human ortholog might employ the same mechanism for tRNA recognition. However, no structural and functional data are available on the human PSTK and the exact molecular detail on how this enzyme might be binding its substrate tRNA is not understood.

An observation that patients suffering from severe autoimmune hepatitis develop autoantibodies against a soluble liver antigen/liver-pancreas (SLA/LP) protein factor (62), which is in turn, often found in complex with Sep-tRNA^Sec^, led to the identification of the terminal synthetic enzyme in the cycle of selenocysteine (63,64). It turned out that SLA/LP is an enzyme termed SepSecS (see above), which promotes the phosphoserine-to-selenocysteine conversion in archaea and eukaryotes. The crystal structure of the human SepSecS-tRNA^Sec^ binary complex, which is the first structure derived from the components of the human selenocysteine synthetic machinery, revealed that SepSecS, just like PSTK, primarily binds the unique 13bp-long acceptor-TΨC arm of tRNA^Sec^ (Figure 2) (49). In contrast to PSTK, however, SepSecS binds the major groove of the acceptor arm and the long variable arm, thus effectively “measuring” the distance between the variable arm and the tip of tRNA^Sec^ (Figure 2). Further, SepSecS and PSTK approach tRNA^Sec^ from opposite sides suggesting that larger multienzyme complexes may be responsible for formation of Selenocysteine. In addition, the structure revealed that SepSecS binds tRNA^Sec^ in a cross-dimer fashion: one homodimer serves as a platform that binds tRNA^Sec^ and orients its CCA end to the catalytic site located in the other SepSecS homodimer. Finally, the results of the structural, mutational, and biochemical studies suggest that SepSecS requires phosphoserine covalently attached to tRNA^Sec^, selenophosphate, and a cofactor, pyridoxal phosphate (PLP), for catalysis (49). Selenophosphate is the major selenium donor in human cells and it is a product of another selenoenzyme, selenophosphate synthetase 2 (SPS2) (65). Selenophosphate is synthesized from ATP and selenide, the end product of selenocysteine degradation promoted by selenocysteine lyase (66). Thus, selenium must first be extracted from the degraded selenoproteins, ingested or endogenous, and turned into selenophosphate before its incorporation into selenocysteine and nascent selenoproteins. SepSecS and SPS2 are positioned at the crossroad between selenium recycling and release, and thus could represent the control points for the synthesis of the entire selenoproteome.

The catalytic functions of SerRS, PSTK, and SepSecS substitute the missing function of the putative SecRS and they serve as distinct checkpoints that ensure that only selenocysteine is paired with tRNA^Sec^. In addition to the elaborate mechanism of selenocysteine synthesis, a separate mechanism evolved to facilitate delivery of Sec-tRNA^Sec^ to the ribosome, the co-translational incorporation of selenocysteine into the nascent protein, and the decoding of the in-frame UGA codon.

## Decoding of the selenocysteine UGA codon

A general elongation factor, EF-Tu in prokaryotes and eEF1A in eukaryotes, delivers to the ribosome all but one aminoacylated tRNA. That sole exception is Sec-tRNA^Sec^. In all domains of life, the delivery of Sec-tRNA^Sec^ to the site of translation requires a specialized elongation factor, SelB in prokaryotes (67,68) and EFsec in eukaryotes (69,70), and a structural element in the selenoprotein mRNA located either within the coding sequence or in the 3′ untranslated region (UTR) (Figure 1). While this general mechanism holds true in all organisms that contain Selenocysteine, some very important differences have been noted between bacteria, archaea, and eukaryotes.

The decoding mechanism for the selenocysteine UGA codon was initially studied using bacterial model systems. It was shown that the process depends critically on a 40 nucleotide-long sequence immediately downstream of the UGA codon, which is termed SElenoCysteine Insertion Sequence (SECIS) (71). The SECIS element forms a hairpin structure, which interacts with a ternary complex composed of SelB, GTP, and Sec-tRNA^Sec^. Each selenocysteine codon in the bacterial selenoprotein mRNA is followed by the SECIS element and thus the bacterial SECIS is often a part of the coding sequence. The crystal structure of the archaeal SelB revealed that the protein contains an N-terminal EF-Tu-like domain and a unique C-terminal domain that contains motifs for binding to SECIS (72). However, the mode of tRNA^Sec^ recognition, the mechanism by which SelB facilitates decoding of the selenocysteine UGA codon and the nature of its interactions with the ribosome are not well understood. In archaea, the SECIS loop is typically in the 3′-UTR of the selenoprotein mRNA and a single SECIS element is capable of coordinating insertion of multiple selenocysteine residues (73,74).

The eukaryotic selenocysteine decoding machinery has another layer of complexity when compared to the bacterial system. For simplicity, we shall discuss primarily the human system. First, human selenoprotein genes typically encode a single selenocysteine residue and they contain a single SECIS element located in the 3′-UTR instead of being a part of the coding sequence (75,76). Exceptions are human selenoprotein P (SelP), which contains 10 selenocysteine residues and two functional SECIS elements, and selenoprotein L (SelL) that contains two selenocysteine residues and one SECIS element (77). Eukaryotic SECIS belongs to the kink-turn family of RNAs comprised of two helical stems separated by an internal loop of 4-18 nucleotides and the distance between the SECIS loop and an in-frame UGA varies significantly among selenoprotein genes (78). One may ask what the purpose of the SECIS element is and why selenocysteine requires this RNA element for its insertion into protein. An explanation would be that binding to the SECIS element localizes both Sec-tRNA^Sec^ and EFsec/SelB near the site of translation, thus effectively increasing their local concentrations. This is important because selenocysteine is encoded by a UGA codon, which otherwise signals the end of translation. The ribosome always stalls at the UGA triplet since under normal circumstances there is no cognate tRNA that reads the UGA codon. This allows for a release factor protein to eventually associate and promote the termination of protein synthesis and the subsequent peptide release from the ribosome. Therefore, if the local concentrations of tRNA^Sec^ and EFsec/SelB are low then the release factors might prematurely abort selenoprotein synthesis. Additionally, the presence of SECIS allows for a more complex regulation of selenocysteine insertion and selenoprotein synthesis as it was already shown in the case of GPx and phospholipid hydroperoxide GPx where the interplay of auxiliary protein factors (eg, SBP2 and eIF4a3) regulates expression levels of these selenoenzymes in response to varying levels of selenium in the liver (79). Further biochemical studies, however, are needed to better understand how different SECIS elements coordinate and regulate selenoprotein synthesis.

Second, in contrast to the prokaryotic SelB, EFsec cannot directly bind a SECIS loop without an auxiliary protein factor, SECIS-binding protein 2 (SBP2) (69,80,81). Mammalian SBP2 is a large protein composed of 854 amino-acid residues. It has been shown that ~ 70% of SBP2 is unstructured in the absence of substrates and binding partners (82). The protein contains three domains: a dispensable N-terminal domain (residues 1-398), a central functional domain (residues 399-517) that is critical for binding to EFsec and ribosome, and a C-terminal RNA binding domain (residues 517-846) responsible for binding to SECIS and ribosome. Invertebrate SBP2 does not contain an N-terminal domain and recently a paralogue of SBP2, SBP2-like protein factor, was shown to participate in selenoprotein synthesis as well (83,84). The RNA binding domain of SBP2 contains a motif that belongs to the L7Ae/L30 family of RNA binding proteins (85), which in turn bind primarily kink-turn RNAs. Thus, it was not surprising that it was shown that SBP2 interacts not only with SECIS, but also with ribosome (85,86). The binding of SBP2 to the 80S ribosome promotes a conformational change in the ribosome, which may facilitate the tRNA accommodation step and/or GTP hydrolysis on EFsec (87). Also, it has been proposed that the binding of SBP2 to SECIS promotes a conformational change in the EFsec-binding pocket of SBP2, which then allows for the interaction between SBP2 and the EFsec:Sec-tRNA^Sec^ binary complex (88). In contrast, SBP2 cannot interact with free EFsec and thus a formation of the productive EFsec:Sec-tRNA^Sec^ complex is essential for selenoprotein synthesis (88). EFsec contains a number of insertions when comparing with SelB. Perhaps the most prominent one is a distinct C-terminal domain IV that might be responsible for binding tRNA^Sec^ and SBP2, and for regulating the GTPase activity of EFsec (89). Structural and biochemical studies on EFsec and its complexes will provide a structural basis for decoding of the selenocysteine UGA codon.

Third, there could be additional, yet unidentified, in-cis regulatory elements in the human selenoprotein mRNAs. For example, it has been shown that the nucleotide sequence immediately downstream of the selenocysteine UGA codon in the mRNA of selenoprotein N (SelN), termed selenocysteine redefinition element (SRE), regulates synthesis of a subset of selenoproteins including SelN (90,91), and mutations in this region have been linked to a series of SEPN1-related myopathies (92).

In spite of the numerous studies, the exact mechanism by which human EFsec and SBP2 bind SECIS and tRNA^Sec^, and how they facilitate decoding of the selenocysteine UGA on the ribosome is not well understood. Further structural, biochemical, and mechanistic studies on the mechanism of decoding of the selenocysteine UGA in humans are thus warranted.

## Disruption of the synthesis and co-translational insertion of selenocysteine causes various diseases in humans

Selenium deficiency, and numerous mutations in selenoproteins and selenoenzymes have been linked to various disorders of the endocrine, central nervous, muscular, cardiovascular, and immune systems in man (93) (Table 1). In contrast, very few mutations in the enzymes responsible for synthesis and co-translational insertion of selenocysteine have been shown to cause any phenotype in humans. This is not entirely surprising considering the essentiality of the selenocysteine cycle for the integrity of the human selenoproteome and for the development of a healthy organism. In other words, given the mouse tRNA^Sec^ knockout mutant displayed embryonically lethal phenotype (47), it is expected that any mutation that has detrimental effect on the structure and function of SerRS, PSTK, SepSecS, SPS2, SBP2, EFsec, or tRNA^Sec^ would most likely completely inhibit selenoprotein synthesis. This, in turn, would yield an embryo incapable of developing into a healthy organism that can reach adulthood and thus such mutations would be selected against in the early stages of embryogenesis. Only in instances in which mutations do not completely impair selenoprotein synthesis, would the phenotype be displayed in some form of disease or disorder. Indeed, recent studies have identified mutations in human SepSecS, SBP2, and the mRNA regulatory elements that cause disorders in humans of different severity and complexity (see below). While mutations affecting selenocysteine synthesis have generally been found to be associated with severe neurological disorders, mutations in the auxiliary components of the decoding apparatus (ie, SBP2, SECIS, and SRE) have been shown, with a few notable exceptions, to cause somewhat milder systemic disorders.

### Mutations in SepSecS cause progressive cerebello cerebral atrophy

**Figure 3:**
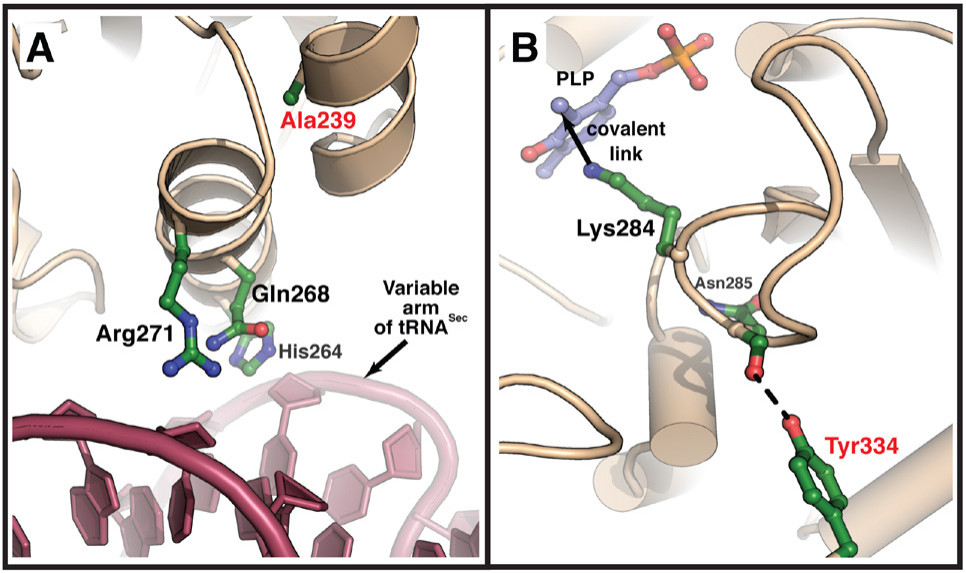
Point mutations in SepSecS give rise to progressive cerebello cerebral atrophy by affecting the tRNA^Sec^-binding pocket and the active-site groove. (**A**) In the first mutant, alanine in position 239 (highlighted in red) in helix α8 is mutated into threonine. This substitution is likely to cause a change in positioning of helices α8 and α9. Because residues in helix α9 (Arg271 and Gln268) interact with the variable arm of tRNA^Sec^ (raspberry), any structural change in this part of the enzyme might reduce the binding affinity of the A239T SepSecS mutant for tRNA^Sec^. (**B**) In the second mutant, a highly conserved tyrosine in position 334 is replaced with cysteine. The Y334C mutation would almost certainly remove an important hydrogen bond between the hydroxyl group in Tyr334 and the backbone carbonyl oxygen in a turn preceding Lys284 to which an obligatory co-factor PLP is covalently attached. Thus, it is likely that the orientation and position of PLP in the Y334C mutant be different than that in the wild-type enzyme. It is plausible that this mutation impairs the catalytic prowess of SepSecS. The enzyme is beige, tRNA^Sec^ is raspberry, and the important amino-acid residues are green stick-and-balls. The ribbon diagrams are based on PDBID 3HL2 (49). Acronyms explained in Figure 1 legend.

Progressive cerebello cerebral atrophy (PCCA) was first identified in nonconsanguineous Jewish Sephardic families of Moroccan and Iraqi ancestry (94). Clinically, PCCA is associated with severe spasticity, profound mental retardation, and progressive microcephaly. Radiological examination revealed that patients exhibit progressive cerebellar atrophy and cerebral atrophy involving both white and gray matter. Most individuals never advanced past the first neurological milestone (smiling) and they rarely lived past 12-13 years of age. Their phenotype most closely resembled pontocerebellar hypoplasia type 2 (PCH2). However, because PCH2 is not associated with progressive cerebellar changes, the search for the cause of this new autosomal recessive syndrome ensued. Sequencing of four inflicted individuals revealed that PCCA is linked to two mutations in the gene encoding SepSecS. It was found that unrelated inflicted individuals of Iraqi Jewish ancestry were homozygous for a mutation in which a nucleotide A1001 is replaced with G. At the protein level, this mutation leads to a substitution of a highly conserved tyrosine residue in position 334 with cysteine (Y334C). On the other hand, inflicted individuals of mixed Iraqi-Moroccan were compound heterozygous for two mutations in the SepSecS gene. Besides the A1001G mutation in one allele, they also carried a G715A mutation in the second allele (termed “Moroccan”). This second mutation leads to a substitution of the conserved alanine 239 with threonine (A239T) in the SepSecS protein. Individuals carrying only one of these mutations exhibit no phenotype, suggesting that one wild-type allele is enough to compensate completely for a single mutation. It was thus hypothesized that the catalytic activity of these mutants is decreased and, indeed, these mutants were not able to complement a strain of *Escherichia coli* lacking the endogenous selenocysteine synthase, SelA (57). Thus, it is likely that the severe neurological phenotype in the inflicted individuals is a consequence of the reduced levels of selenoproteins. Although the selenium content of the brain is not particularly high, selenoprotein P (SelP) delivers selenium preferentially to the brain at the expense of other organs under selenium deficiency, which suggests that the constant selenium content in the brain is essential for normal development. This is in agreement with the observations that mice in which either SelP (95) or its receptor, ApoER2 (96), were knocked out, displayed ataxia and seizures (97,98) and that those in which tRNA^Sec^ was deleted from neurons had neurodevelopmental and degenerative phenotype in the cerebellum, hippocampus, and cerebral cortex (99). However, it is not clear how these mutations affect the structure and function of SepSecS. It is likely that the insertion of Thr instead of Ala239 causes steric clashes between two α helices that form a binding pocket for the variable arm of tRNA^Sec^ (Figure 3A). Thus, the expectation would be that, if properly folded, the A239T mutant of SepSecS would bind tRNA^Sec^ with lesser affinity compared to the wild type protein, whereas its catalytic function would be unaffected. On the other hand, the Y334C mutation is thought to affect the architecture of the active site and particularly the positioning of the co-factor pyridoxal-5-phosphate (PLP). The hydroxyl moiety of Tyr334 forms a hydrogen bond with the backbone atoms of the turn that carries Lys284 to which PLP is covalently attached (Figure 3B). Because Cys is much shorter than Tyr, the hydrogen bond would not be formed and PLP would be repositioned. Therefore, it is likely that this mutant would bind tRNA^Sec^, but it would not be capable of catalyzing the phosphoserine-to-selenocysteine conversion with the same efficiency as the wild type enzyme.

Two completely different mutations in SepSecS than those described in the PCCA study have been identified in three unrelated families in the Finnish population (personal communication with Dr Henna Tyynismaa). Compound heterozygous individuals exhibit a similar neurological phenotype as those with PCCA; the inflicted individuals display severe mental retardation, progressive cerebellar and cerebral atrophy, microcephaly, extreme spasticity, and other features characteristic of that disorder (personal communication with Dr Henna Tyynismaa). Additional symptoms have been observed, which suggest that this may be a distinct disorder related to SepSecS mutations.

### Human diseases caused by mutations in the selenocysteine UGA decoding apparatus, SBP2 and SECIS/SRE

Numerous mutations have been found in the human SBP2 gene leading to multisystemic disorders. Also, links between human disorders and two mutations in in-cis regulatory elements (ie, SECIS and SRE) that coordinate decoding of the selenocysteine UGA codon have been characterized.

Refetoff et al were first to report on three mutations in SBP2 linked to abnormal thyroid hormone metabolism (100). In case of the Bedouin Saudi family, the affected individuals carried a homozygous missense mutation in exon 12 which resulted in the R540Q amino acid substitution in the SBP2 protein. The inflicted individuals had abnormal thyroid function tests: elevated blood thyrotropin (TSH), elevated T_4_ levels, and reduced T_3_ levels. Also, the levels of iodothyronin deiodinase 2 (DIO2), which is responsible for producing the most active form of thyroid hormone (101), were reduced and the patients displayed delayed bone growth resulting in a short stature. In a subsequent study it has been shown that the R540Q mutant of SBP2 had reduced affinity for a subset of SECIS RNAs including those for DIO1, DIO2, and GPx1 (102). Given that SBP2 preferentially binds certain SECIS elements (103), it has been suggested that the R540Q mutation affected the hierarchy of selenoprotein synthesis in general, and that of the enzymes of thyroid hormone metabolism in particular. A similar phenotype observed in an unrelated family of Irish origin was caused by a compound heterozygous defect in SBP2 (100). The patient’s inherited paternal allele carried a nonsense mutation K438X in exon 10, which resulted in a truncated protein devoid of any function, and a maternal allele with a mutation IVS8ds +29G→A, which created an alternative splice site in the SBP2 transcript that caused a frameshift and a complete change in protein sequence. The abnormally spliced transcripts represented ~ 52% of the transcripts generated from the maternal allele. Consequently, the patient had ~ 25% of normal SBP2 transcripts and presumably that same level of functional protein compared to a normal and healthy individual. In both instances, it was shown that the activity of glutathione peroxidase in serum (GPx-3) was reduced and that serum levels of SelP and selenium were significantly reduced compared to healthy individuals.

Subsequently, Refetoff et al identified yet another mutation in the SBP2 gene in a family of African origin (104). In this peculiar case, the patient was homozygous to a R128X mutation, which was supposed to yield a severely truncated and dysfunctional protein factor. However, the phenotype was mild and reminiscent of the previously described partial SBP2 deficiency (100) albeit with normal TSH levels. Further examination of the expression profile generated from the SBP2 minigene revealed that at least three ATG codons downstream of the regular start codon support synthesis of SBP2. The N-terminal domain of SBP2 is dispensable and the truncated SBP2 constructs supported selenoprotein expression at a somewhat attenuated level, but which was sufficient to prevent expression of a more severe phenotype. Similar phenotype to the R128X was also described in Japan, but the cause was a distinct compound heterozygous defect in SBP2 (105). In this case, the maternal copy carried a Q79X nonsense mutation and the paternal allele had a mutation that caused a frame shift and insertion of the stop codon after 48 amino acids. The Q79X allele, just like R128X, supposedly yields a truncated and functional SBP2 by utilizing downstream ATG codons, whereas the mRNA derived from the second copy is targeted to the nonsense-mediated mRNA decay.

More recently, researchers from Brazil reported on a 12-year old girl with a syndrome of abnormal thyroid metabolism, delayed bone maturation, congential myopathy, and impaired mental and motor coordination development (106), which was a result of yet another compound heterozygous defect in SBP2. The patient inherited a paternal allele with a R120X nonsense mutation and a maternal allele with another nonsense mutation, R770X. While the allele carrying the R120X mutation could still support expression of the truncated but functional protein (like R128X and Q79X), the R770X substitution yields dysfunctional SBP2 because the truncation occurs in the C-terminal domain critical for binding to SECIS and EFsec. Because only one allele could support synthesis of the somewhat functional SBP2, the phenotype was more severe than in the case of the R128X homozygous mutation. The patient had reduced levels of DIOs and undetectable levels of SelP, which are perhaps responsible for growth defects and neurological abnormalities. Finally, the patient exhibited myopathy similar to that observed in patients harboring mutations in SEPN1 gene suggesting that R770X mutation perhaps affected the expression of selenoprotein N (SelN).

Perhaps the most complex multisystem disorder caused by mutations in the SBP2 gene was described by Chatterjee et al (107). Two patients had a very complex clinical picture: abnormal thyroid metabolism, low levels of selenium, reduced synthesis of selenoproteins including SelP and GPx3, azoospermia and spermatogenesis failure (infertility), axial muscular dystrophy, myopathy, skin photosensitivity, and abnormal immune cell function. In addition, increased reactive oxygen species production, accelerated telomere shortening, membrane lipid peroxidation and DNA damage were observed at the cellular level. Both patients were compound heterozygous for particular yet different mutations in SBP2. The first patient inherited a paternal allele in which a premature stop codon was introduced in exon 5 as a result of a frameshift mutation. The resulting protein is likely a truncated but functional isoform of SBP2. The maternal allele contained a splicing defect between exons 6 and 7, which presumably yields SBP2 devoid of the functionally important C-terminal domain. The second patient carried a maternally inherited C691R mutation and a paternally inherited splicing defect in SBP2. While the functional protein could not be expressed from the paternal allele, the maternal allele could give rise to a full-length C691R mutant of SBP2. The C691R mutation not only affects the RNA binding of SBP2, but also targeting of this mutant to the proteasome is enhanced thus reducing the overall level of SBP2.

Besides mutations in SBP2, two mutations in the regulatory elements of the selenoprotein mRNAs have been identified to cause disease in humans. It has been shown that the apical helix of the SECIS element of the Sep15 mRNA contains a single nucleotide polymorphism 1125G→A (108). Diamond et al have shown that frequency of this SNP varies with ethnicity and that might contribute to cancer risk (109). However, it is still not clear how this change in the region of SECIS not critical for SBP2 binding might affect selenoprotein synthesis. Further, Guicheney et al have shown for the first time that a single homozygous mutation in the SECIS element causes a disease in humans. In particular, a 17195T→C substitution in the 3′-UTR of the gene encoding the SelN protein causes SEPN1-related myopathy characterized with marked muscle weakness and a significant restrictive respiratory insufficiency (110). The mutation, which affects the 5′ U in the non-Watson-Crick quartet in the core of the SECIS stem, impairs SelN expression in vivo and SBP2 binding to SECIS in vitro. More recently, Howard et al identified a series of mutations (G463V, heterozygous; R469W, homozygous; R469Q, homozygous; R466Q, heterozygous) within the SRE element that cause SEPN1-related myopathies of various severities (92). All patients exhibited congenital muscle weakness, spinal rigidity, scoliosis, and respiratory insufficiency. While most patients underwent spinal fusion and required nasal ventilation, they remained ambulant. Two patients developed severe phenotypes and died at the age of 5.5 and 7 years. The SRE element is immediately adjacent to the UGA codon and it regulates the expression of a subset of selenoproteins including SelN. It has been shown that the mutations affect the secondary structure of SRE, dampen the efficiency of co-translational insertion of selenocysteine, and reduce levels of the SelN mRNA and the SelN protein in muscle.

## Concluding remarks

**Figure 4:**
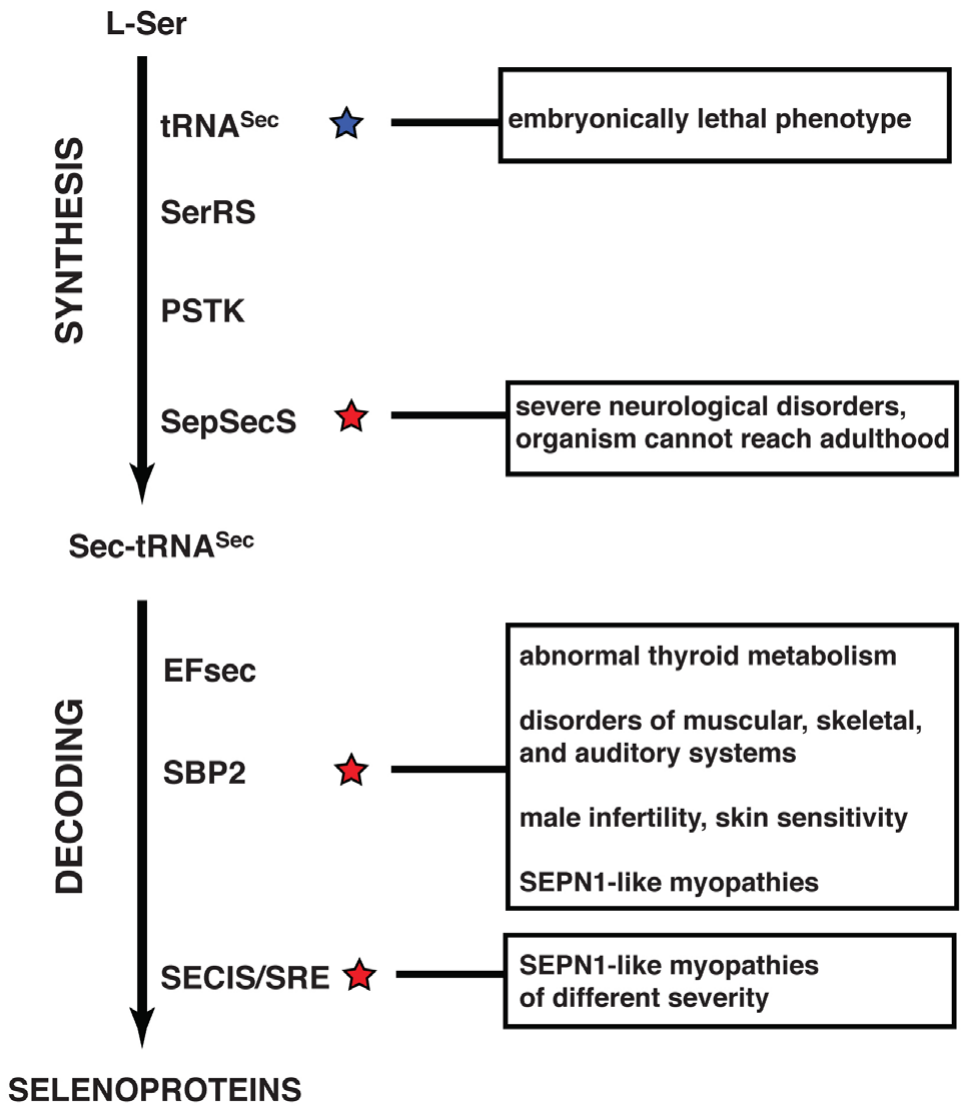
Mutations in components of the synthetic and decoding apparatus of selenocysteine have distinct effects on health of the human organism. A normal cycle supports synthesis of suitable levels of selenoproteins. Mutations (red star) in various components have been shown to cause disorders in humans. In particular, point mutations in SepSecS lead to severe neurological disorders in which the inflicted individuals cannot reach adulthood. Additionally, point mutations in the decoding apparatus (SBP2, SECIS, and SRE) cause a variety of disorders that are rarely lethal. The essentiality of the selenocysteine cycle has also been supported by the embryonic lethal phenotype of the mouse tRNA^Sec^ knockout mutant (blue star). Presumably, these mutations attenuate selenoprotein synthesis to a different degree, hindering development of the individual with differing levels of severity. Acronyms explained in Figure 1 legend.

Selenocysteine, the 21st amino acid, is absolutely essential for human health and survival. The most prominent members of the human selenoproteome regulate thyroid metabolism, remove reactive oxygen species and protect the cell from oxidative damage, maintain the cellular redox balance and participate in redox reactions, regulate signaling cascades, promote protein folding, and maintain selenium homeostasis. Mutations in selenoproteins and selenium deficiency have long been linked to a variety of human disorders, some of which have been treated by dietary selenium supplements. Synthesis of selenoproteins and the integrity of the selenoproteome, however, depend critically on formation and accurate co-translational incorporation of selenocysteine. In contrast to the 20 standard amino acids, selenocysteine is synthesized from a serine precursor through a series of reactions taking place on its unique tRNA. Moreover, selenocysteine is targeted to the site of translation by a specialized elongation factor, an *in-trans* protein factor, and *in-cis* elements in the selenoprotein mRNA, which coordinate decoding of the selenocysteine UGA codon. Emerging evidence accumulated over the last two decades convincingly argues that the integrity of the selenocysteine-synthetic and decoding apparatus is essential to the development of a normal and healthy human organism (Figure 4). Mutations in the terminal synthetic enzyme, SepSecS, cause extremely detrimental neurological disorders in which the inflicted individuals do not develop past puberty. In addition, mutations in the components of the decoding apparatus (ie, SBP2, SECIS, SRE) cause disorders of endocrine, muscular, skeletal, and cardiovascular systems. With the exception of two cases, phenotypes of the decoding system are generally milder than the ones linked to the malfunctioning synthetic cycle. However, it remains to be seen how these mutations affect the structure and function of the mutated proteins, enzymes, and regulatory mRNA elements, and if such information could be rationally used in remedying at least some of the disorders. It is reasonable to expect that more disorders linked to selenocysteine cycle will be uncovered, in particular, providing possible insight into neurological disorders of unknown etiology. Also, because of its essentiality for survival of all human cells, in particular, life-threatening cancer cells, the selenocysteine machinery should at least be considered as a potential therapeutic target. Finally, it is important to mention that several protozoan pathogens such as *Trypanosoma*, *Plasmodium,* and *Leishmania* depend on selenium, suggesting that structural and functional studies on the selenocysteine cycle in these organisms could reveal differences that could, in turn, be employed for design of novel therapies. It has become abundantly clear that research centered on selenium and its role in development and maintenance of the human organism will continue to grow in importance as more selenocysteine related pathologies are uncovered. We are just beginning to understand the wide-spread dependence on selenium for a majority of living organisms, in particular the entire animal kingdom.
